# Patterns of Workplace Absenteeism Across Levels of Virological Failure Among Employed Adults Co-Infected with HIV and Pulmonary Tuberculosis in the Govan Mbeki Sub-District, South Africa: A Retrospective Record Review

**DOI:** 10.3390/healthcare14183092

**Published:** 2026-09-19

**Authors:** Tinyiko Mabunda, Tanusha Singh

**Affiliations:** Department of Environmental Health, Faculty of Health Sciences, University of Johannesburg, Doornfontein, Johannesburg 2028, Gauteng, South Africa

**Keywords:** HIV, pulmonary tuberculosis, virological failure, viral load, workplace absenteeism, mine workers, occupational health, South Africa

## Abstract

**Highlights:**

**What are the main findings?**

- Among participants selected for virological failure, 63.3% had high-level virological failure (≥10,001 copies/mL); this percentage describes the distribution within the selected cohort and is not a prevalence estimate for the source population. Workplace absenteeism averaged 10.05 sick-leave days over the preceding three months; 129 participants (45.1%) were classified as having high absenteeism (11–15 days) and 157 (54.9%) as having lower absenteeism (5–10 days). Viral-load category was not independently associated with high absenteeism.

**What are the implications of the main findings?**

- The concurrent occurrence of high-level virological failure and absenteeism in this selected employed cohort warrants attention, but the present analysis does not establish that one caused or predicted the other.
- Integrated HIV/TB and occupational health services should consider treatment access and continuity of care, while recognising that the determinants of absenteeism were incompletely measured in this study.

**Abstract:**

**Background/Objectives**: Human immunodeficiency virus (HIV) and tuberculosis (TB) remain major public health challenges in sub-Saharan Africa, which bears the highest burden of HIV/TB co-infection. Evidence is limited on whether the severity of virological failure is associated with workplace absenteeism among employed adults with HIV/PTB co-infection. This study examined workplace absenteeism across two viral-load categories among employed adults with documented virological failure in the Govan Mbeki sub-district. **Methods**: A retrospective record review with secondary data analysis was conducted among 286 employed mine workers with HIV/PTB co-infection and a viral load ≥1000 copies/mL after at least six months of antiretroviral therapy (ART). Clinical data were extracted from medical, laboratory, and TB-treatment records and linked to workplace sick-leave registers; limited participant contact was used only to clarify essential missing information. The analytic dataset had an observed minimum viral load of 1200 copies/mL. Viral load was categorised as 1200–10,000 versus ≥10,001 copies/mL. Absenteeism was the number of recorded sick-leave days during the preceding three months and, for logistic regression, was classified as low (5–10 days) versus high (11–15 days). Multivariable logistic regression was adjusted for age, sex, education, and structural/socioeconomic barrier score. **Results:** Mean absenteeism was 10.05 days (SD = 2.44). Of 286 participants, 181 (63.3%) had a viral load ≥10,001 copies/mL and 105 (36.7%) had a viral load of 1200–10,000 copies/mL. High absenteeism occurred in 129 participants (45.1%) and lower absenteeism in 157 (54.9%). Viral load category was not associated with high absenteeism after adjustment (aOR = 1.21, 95% CI: 0.76–1.92; *p* = 0.432). The model was not statistically significant (χ^2^ = 6.41, df = 5, *p* = 0.269), explained 3.0% of the variance (Nagelkerke R^2^ = 0.030), and had an acceptable Hosmer–Lemeshow fit (*p* = 0.913). **Conclusions**: Within this selected employed cohort with virological failure, high-level virological failure and absenteeism were both common, but no evidence of an independent association between viral-load category and high absenteeism was detected. The cross-sectional temporal structure, healthy-worker selection, limited exposure contrast, and residual confounding constrain causal interpretation. Integrated public health and workplace interventions are suggested to improve both treatment outcomes and workforce participation in high-burden settings.

## 1. Introduction

Human immunodeficiency virus (HIV) and tuberculosis (TB) remain among the most significant infectious diseases globally and continue to pose a major public health challenge, particularly in sub-Saharan Africa [1]. The epidemiological overlap between these two conditions is well established, with HIV significantly increasing susceptibility to TB infection and progression from latent to active disease. Globally, an estimated 10 million people develop TB annually, with a substantial proportion occurring among individuals living with HIV [1]. In turn, TB remains the leading cause of death among people living with HIV (PLHIV) worldwide [2]. South Africa carries one of the highest burdens of HIV/TB co-infection globally [1].

The interaction between these diseases is bidirectional: HIV-induced immunosuppression increases the risk of TB, while TB infection may accelerate HIV disease progression through immune activation and increased viral replication [1,2,3]. Virological failure, typically defined as a persistent viral load ≥1000 copies/mL despite antiretroviral therapy (ART), represents a critical concern in HIV management [2]. Among individuals co-infected with TB, achieving viral suppression may be particularly difficult due to increased pill burden, treatment fatigue, drug interactions, and frequent healthcare visits. Data suggest that TB co-infection is associated with higher rates of virological failure, particularly among individuals receiving antiretroviral therapy [3]. Beyond virological outcomes, HIV/TB co-infection is also linked to broader clinical consequences, including increased mortality and greater treatment complexity [4].

According to the World Health Organisation (WHO), approximately 7.8 million people were living with HIV in South Africa in 2023, while an estimated 280,000 new TB cases were reported annually [1]. Furthermore, approximately 50% of individuals diagnosed with TB in South Africa are co-infected with HIV [1]. Despite widespread access to antiretroviral therapy (ART), virological failure and unsuppressed viral load remain major public health concerns, particularly among individuals co-infected with HIV and TB [2,3]. The persistence of unsuppressed viral load not only affects individual treatment outcomes but may also have broader social and economic implications through its impact on daily functioning and work participation.

Beyond clinical outcomes, HIV and TB have important socioeconomic implications [5,6,7]. Individuals affected by these conditions are often within the economically productive age group and may experience disruptions to employment due to illness episodes, treatment side effects, and healthcare utilisation [5,6,7,8,9]. Chronic infectious diseases have been shown to reduce labour productivity and workforce participation, particularly in settings where employment depends on physical capacity and consistent attendance [5,6,7]. In occupational environments such as mining, construction, and industrial labour, these effects may be amplified [1].

Workplace absenteeism is a key indicator of functional health status and productivity [1]. Among individuals living with HIV and TB, absenteeism may result from a combination of clinical and structural factors, including disease severity, treatment burden, access to healthcare, and workplace conditions [3,4,5,6,7,8,9,10]. Structural barriers such as transport challenges, employment constraints, and healthcare access limitations have been identified as important determinants of HIV treatment outcomes and may indirectly impact work participation [8,9,10,11]. Previous studies have reported increased absenteeism, reduced productivity, and income loss among individuals living with HIV or TB; however, these studies have largely focused on disease status, treatment adherence, or disability rather than virological outcomes such as viral load suppression [5,6,7].

Previous studies have documented work disruption, treatment adherence, disability, and labour-market consequences among people affected by HIV or TB [1,2,3,4,5,6,7,8,9,10]. The narrower question addressed here is different: whether, among employed adults already meeting criteria for virological failure and co-infected with PTB, the degree of viral-load elevation is associated with recorded workplace absenteeism. Evidence directly linking viral-load strata to objectively recorded sick-leave days in employed HIV/PTB co-infected populations is limited. The study therefore does not claim that work-related determinants of HIV/TB outcomes are unexplored; rather, its contribution is the linkage of routine virological measurements with workplace absenteeism records in a high-burden occupational setting.

The relationship between the severity of virological failure and workplace absenteeism remains uncertain. Attendance may reflect TB morbidity, treatment burden, healthcare utilisation, workplace policies, and other clinical or psychosocial factors in addition to viral load. This study therefore aimed to assess whether high-level virological failure (≥10,001 copies/mL), compared with moderate virological failure (1200–10,000 copies/mL), was associated with high workplace absenteeism among employed adults with HIV/PTB co-infection in the Govan Mbeki sub-district. Since all analysed participants had virological failure, the study does not compare virally suppressed with unsuppressed individuals and cannot estimate the effect of virological failure itself on absenteeism.

## 2. Materials and Methods

### 2.1. Study Design

A retrospective record review with secondary data analysis was undertaken. Existing clinical, laboratory, TB-treatment, and workplace absenteeism records were linked for eligible employed adults. The analysis was cross-sectional in the sense that viral-load status and recent absenteeism were evaluated within the same study period; however, the design was not a prospective cross-sectional survey. Limited participant contact occurred only when essential information required clarification and did not constitute prospective recruitment or a primary interview survey. Accordingly, the design supports an assessment of association but not temporal direction or causality.

### 2.2. Study Setting

The study was conducted in the Govan Mbeki sub-district, South Africa, an area characterised by substantial industrial and mining activity and a large working population. According to district health records, approximately 38,782 individuals in the sub-district were living with HIV (Mpumalanga Department of Health, 2023, unpublished data). Of these, 2005 were co-infected with PTB, and 1093 had documented unsuppressed viral load levels greater than 1000 copies/mL. Data were collected from five healthcare facilities and two hospitals within the sub-district, as well as from workplace absenteeism registers in participating mining companies.

### 2.3. Study Population

The study population consisted of formally employed adults working in participating mining companies who were co-infected with HIV and PTB, had documented virological failure, and received treatment at participating healthcare facilities in the Govan Mbeki sub-district. The term ‘employed adults’ is used because occupational category and shift pattern were not available in sufficient detail for stratified analysis.

### 2.4. Inclusion and Exclusion Criteria

Records were eligible if they related to adults aged ≥18 years with confirmed HIV infection and PTB, documented viral load ≥1000 copies/mL after at least six months of uninterrupted ART, and formal employment during the three-month absenteeism observation window. The six-month ART requirement was used to ensure sufficient treatment exposure before classifying virological failure in accordance with the study protocol and national guidance [2]. A separate minimum duration of employment was not specified beyond being employed during the observation window; this is acknowledged as a limitation. Records were excluded if variables required for analysis were incomplete or if duplicate records were identified.

### 2.5. Sample Size and Sampling Procedure

Sample size was calculated using Epi Info™ software version 7.2.5 (Centers for Disease Control and Prevention, Atlanta, GA, USA) [12] based on a finite population of 1093 individuals with documented virological failure in the Govan Mbeki sub-district. Using a 95% confidence level, a 5% margin of error, finite-population correction, and a 38.4% prevalence estimate, the required sample size was 286 records. A total of 315 records were consequently screened from the available record pool, of which 286 met the eligibility and completeness criteria and were included in the final analysis. Given the final analytic comparison between moderate and high-level virological failure, a detectable-effect assessment was also conducted. Based on the observed group sizes (105 with moderate and 181 with high-level virological failure) and the observed high-absenteeism proportion in the moderate group (43/105), the sample provided approximately 80% power at a two-sided α = 0.05 to detect an odds ratio of approximately 2.0 (or approximately 0.48 in the opposite direction). Therefore, the study was adequately positioned to detect relatively large associations, while smaller associations may not have been detected.

### 2.6. Data Collection Procedures

#### Secondary Data Extraction

The record-identification and eligibility-screening phase occurred from 16 to 24 October 2025. Clinical and absenteeism data were then extracted from paper and electronic medical records, TB-treatment folders, viral-load laboratory reports, and workplace attendance/sick-leave registers from 27 October to 12 December 2025. Data cleaning and statistical analysis were conducted from 12 January to 27 February 2026; these later dates were not part of data collection. Where an essential item could not be resolved from linked clinical or administrative records, limited clarification was sought from the participant after consent. The study was therefore a retrospective record review supplemented by limited clarification, not a structured interview survey.

For the limited instances in which participant clarification was required, trained bilingual research assistants explained the study and consent procedures in participants’ preferred languages. No additional structured interview dataset was created. Research assistants were bound by the same confidentiality obligations as the research team and were proficient in at least two commonly spoken local languages.

### 2.7. Study Variables

#### 2.7.1. Outcome Variable

Workplace absenteeism was defined as the number of recorded sick-leave days during the three months preceding the index data extraction. A three-month window was specified a priori to capture recent absence while limiting temporal drift and record-retrieval variability. Absenteeism was retained as a continuous descriptive measure and was also grouped as 5–7, 8–10, and 11–15 days. For the prespecified binary logistic regression, lower absenteeism was defined as 5–10 days (*n* = 157; 54.9%) and high absenteeism as 11–15 days (*n* = 129; 45.1%), corresponding to the upper observed category. This threshold is study-specific rather than a validated clinical cut-off. Scheduled workdays, shift patterns, and occupational categories were not available consistently, so the measure represents absolute sick-leave days rather than a proportion of scheduled work time.

#### 2.7.2. Exposure Variable

The primary exposure variable was the degree of virological failure. Eligibility required a viral load ≥1000 copies/mL; however, the lowest viral-load value among records retained in the final analytic dataset was 1200 copies/mL, and no analysed record fell between 1000 and 1199 copies/mL. Thus, 1200 copies/mL was the observed analytic minimum rather than an additional recruitment threshold. For the primary analysis, viral load was categorised as moderate virological failure (1200–10,000 copies/mL) and high-level virological failure (≥10,001 copies/mL). The ≥10,001 cut-point was a study-defined severity threshold used in the original analysis and should not be interpreted as a WHO-defined category. WHO guidance supports ≥1000 copies/mL as the threshold for virological failure; the additional 10,000-copy split is therefore acknowledged as an analytic categorisation and a limitation.

The final analytic dataset contained complete observations for the variables used in the analyses (*n* = 286); therefore, no participant was excluded from the fitted models because of missing analytic variables. Of 315 records initially screened, 286 met the eligibility and completeness criteria. Individual-level data for the excluded records were not retained in the analytic dataset; consequently, a formal comparison of included and excluded records could not be undertaken, and selection at the record-screening stage cannot be excluded.

### 2.8. Data Management

Data were entered, cleaned, and analysed using IBM SPSS Statistics Version 30 (IBM Corporation, Armonk, NY, USA). The dataset was screened for missing values, outliers, and coding inconsistencies before analysis. Missing data were minimal (<5%) and complete-case analysis was used for variables required in the regression. Continuous variables were checked using distributions and boxplots; extreme values were verified against source records and retained where plausible. Categorical coding was cross-checked against source records. Electronic data were password-protected devices and hard copies were stored in locked cabinets accessible only to the researcher team.

The socioeconomic barrier score range of 0–2 (mean 0.94, SD 0.99) included: 148 participants scored 0, 8 scored 1, and 130 scored 2. The analytic dataset also contained separate barrier indicators for employment-related, housing, food, and other socioeconomic barriers. A separate transport-challenge variable was invariant (286/286 coded present) and therefore provided no discriminatory information. Because the observed score is a narrow ordinal measure, it was entered categorically in the additional count-model analysis. Its study-specific construction and limited variability restrict interpretation and generalisability.

### 2.9. Statistical Analysis

Descriptive statistics were used to summarise participant characteristics. Categorical variables were reported as frequencies and percentages, while continuous variables were summarised using means and standard deviations. Chi-square (χ^2^) tests were used to examine associations between categorical variables, and an independent-samples *t*-test was used to compare mean sick-leave days by sex.

Workplace absenteeism was recorded as the number of sick-leave days during the preceding three months. For the categorical analysis, lower absenteeism was defined as 5–10 sick-leave days and high absenteeism as 11–15 sick-leave days. Binary logistic regression was used to estimate the association between high-level versus moderate virological failure and high versus lower absenteeism. The multivariable model adjusted for age, sex, education level, and the socioeconomic barrier score. These covariates were selected a priori because they were consistently available in the linked records and were considered plausible determinants of treatment outcomes and work attendance. Adjusted odds ratios (aORs) with 95% confidence intervals (CIs) and two-sided *p*-values were reported. Model fit was assessed using the omnibus test, Nagelkerke R^2^, and the Hosmer–Lemeshow goodness-of-fit test [13,14].

A precomputed socioeconomic barrier score was available in the analytic dataset. The observed score ranged from 0 to 2 (mean = 0.94, SD = 0.99), with 148 participants scoring 0, eight scoring 1, and 130 scoring 2. Separate variables captured employment-related, housing, food, and other socioeconomic barriers. The transport-challenge variable was invariant in the analytic dataset and therefore did not discriminate barrier burden. Because the exact derivation of the precomputed composite score could not be reconstructed with sufficient certainty from the analytic dataset alone, no constituent formula or weighting scheme was inferred retrospectively. The score was retained as recorded in the original analysis. Given its narrow ordinal distribution, it was entered categorically in the additional count-based analysis, thereby avoiding an assumption of a linear effect across successive score values.

Because dichotomising absenteeism may result in loss of information and statistical power, absenteeism was additionally analysed in its original count form. The distribution showed underdispersion rather than overdispersion (mean = 10.05 days; variance = 5.97; variance-to-mean ratio = 0.59). Consequently, negative binomial regression was not selected as the primary count model. Poisson regression with robust standard errors was fitted to estimate incidence rate ratios (IRRs) for sick-leave days, adjusting for age, sex, education level, and the socioeconomic barrier score entered categorically. Adjusted IRRs with 95% CIs and two-sided *p*-values were reported.

Because the ≥10,001 copies/mL threshold was a study-defined analytic cut-point rather than an established clinical severity threshold, an additional sensitivity analysis modelled viral load as a continuous variable after log10 transformation. This analysis assessed whether categorisation of viral load at 10,000 copies/mL obscured information contained in the continuous measure. The resulting IRR represents the relative change in the expected number of sick-leave days associated with a 10-fold increase in viral load.

All statistical tests were two-sided, with statistical significance set at *p* < 0.05. The regression analyses were explanatory rather than predictive. Potential residual confounding was considered because several clinically and occupationally relevant variables were not consistently available in the linked records.

### 2.10. Pilot Study

A pilot study was conducted using ten records outside the main study sample. The purpose of the exercise was to assess the feasibility, clarity, and completeness of the structured data extraction tool. The pilot study assisted in identifying minor inconsistencies in data recording and variable categorisation. Necessary adjustments were made to improve the clarity and organisation of the data extraction tool. Data obtained during the pilot study were not included in the final analysis.

### 2.11. Reliability and Validity

Reliability was strengthened through the use of a structured and standardised data extraction tool aligned with the study objectives and variables identified in previous literature [15]. The pilot study demonstrated that the tool was appropriate for consistently extracting demographic, clinical, and occupational data across participant records. Standardised data collection procedures were followed throughout the study. Data quality checks, cross-checking of records, and verification procedures were conducted to minimise inconsistencies and improve the reliability of the collected data.

Internal validity was supported using clearly defined inclusion criteria, standardised viral load thresholds, and multivariable adjustment for potential confounding. External validity may be limited to similar occupational settings in high HIV/TB burden regions.

### 2.12. Ethical Considerations

Ethical approval was obtained from the University of Johannesburg Faculty of Health Sciences Research Ethics Committee (REC-3803-2025), with permissions from relevant district health authorities, facilities, and participating workplaces. The approved protocol required written informed consent from living participants whose identifiable clinical and occupational records were linked for the study and/or who could be contacted for clarification. Only records meeting the approved consent and eligibility requirements were included in the analytic dataset. Participant contact was limited to consent/clarification where required and was not used to create a prospective survey dataset. Study codes replaced direct identifiers in the analytic file, and linkage information was access-restricted. Participants could withdraw before deidentification and analysis without penalty. All procedures were conducted in accordance with ethical principles for research involving human participants [16].

## 3. Results

This section presents the demographic, clinical, and occupational characteristics of the study participants, followed by the bivariate and multivariate analyses.

### 3.1. Participant Socioeconomic Characteristics

A total of 286 participants were included in the final analysis. The sample was almost evenly distributed by sex, with 141 males (49.3%) and 145 females (50.7%). Participants were predominantly within the economically active age range (Table 1). The largest age category was 30–39 years (31.1%), followed by 40–49 years (27.6%), 20–29 years (23.4%), and 50 years or older (17.8%). Educational attainment varied, with secondary education being the most common level achieved (36.4%), followed by primary education (32.2%) and tertiary education (30.4%). Only 1.0% of participants reported no formal schooling. All participants were permanently employed at the time of data collection.

All 286 participants had confirmed diagnoses of HIV/PTB co-infection and met the study eligibility criterion for virological failure. The observed minimum viral load in the analytic dataset was 1200 copies/mL. High-level virological failure (≥10,001 copies/mL) occurred in 181 participants (63.3%), while 105 (36.7%) had moderate virological failure (1200–10,000 copies/mL). These percentages describe the distribution of viral-load severity within a cohort selected for virological failure and are not prevalence estimates. Mean sick leave was 10.05 days (SD = 2.44; range 5–15). For the binary analysis, 129 participants (45.1%) had high absenteeism (11–15 days) and 157 (54.9%) had lower absenteeism (5–10 days) (Table 1).

### 3.2. Structural and Socioeconomic Barriers

Employment-related barriers were recorded for 138 participants (48.3%), while the separate “other socioeconomic barrier” indicator was present for 132 (46.2%). Housing instability and food insecurity showed no variability, and the transport-challenge variable was invariant (286/286 coded present). The precomputed socioeconomic barrier score had a mean of 0.94 (SD 0.99) and an observed range of 0–2, with 148 participants scoring 0, 8 scoring 1, and 130 scoring 2 (Table 2).

### 3.3. Bivariate Analysis

Cross-tabulation of viral-load category and binary absenteeism showed that among participants with moderate virological failure, 43/105 (41.0%) had high absenteeism and 62/105 (59.0%) had lower absenteeism, compared with 86/181 (47.5%) and 95/181 (52.5%), respectively, among participants with high-level virological failure. This association was not statistically significant, χ^2^(1) = 1.16, *p* = 0.282. Similarly, no statistically significant associations with absenteeism were observed for sex [χ^2^(1) = 0.73, *p* = 0.392], age group [χ^2^(3) = 1.19, *p* = 0.756], or employment-related barriers [χ^2^(1) = 2.21, *p* = 0.138]. An independent-samples *t*-test also showed no statistically significant difference in mean sick-leave days between males and females [t(284) = −0.65, *p* = 0.518].

### 3.4. Logistic Regression Analysis of Factors Associated with Workplace Absenteeism

Binary logistic regression was then performed to assess crude associations between independent variables and absenteeism (Table 3). Viral load category (OR = 1.305; 95% CI: 0.803–2.123; *p* = 0.283) was not significantly associated with absenteeism. Similarly, age (OR = 1.009; 95% CI: 0.986–1.032; *p* = 0.466), sex (OR = 1.226; 95% CI: 0.769–1.954; *p* = 0.393), education (OR = 0.929; 95% CI: 0.567–1.523; *p* = 0.772), and socioeconomic barrier score (OR = 0.840; 95% CI: 0.662–1.066; *p* = 0.152) were not significantly associated with absenteeism. All corresponding 95% CIs included unity.

A multivariable logistic regression analysis was conducted to examine the association between viral load category and workplace absenteeism, adjusting for age, sex, education level, and socioeconomic barrier score (Table 3). The overall model was not statistically significant (χ^2^ = 6.407, df = 5, *p* = 0.269), indicating that the included covariates were not jointly associated with high absenteeism at the specified significance level. The model had limited explanatory capacity (Nagelkerke R^2^ = 0.030). The Hosmer-Lemeshow test was non-significant (*p* = 0.913), indicating acceptable model fit. High-level versus moderate virological failure was not significantly associated with high absenteeism after adjustment (aOR = 1.205; 95% CI: 0.757–1.917; *p* = 0.432). Age (aOR = 0.999; 95% CI: 0.970–1.030; *p* = 0.934), sex (aOR = 1.170; 95% CI: 0.730–1.880; *p* = 0.499), education level (aOR = 0.807; 95% CI: 0.520–1.250; *p* = 0.374), and socioeconomic barrier score (aOR = 0.808; 95% CI: 0.636–1.027; *p* = 0.081) were also not statistically significant (Table 3).

**Table 3:** Logistic regression analysis of factors associated with high absenteeism.

Variable	Category	Crude Odds Ratio			Adjusted Odds Ratio		
		OR	95% CI	*p*-Value	aOR	95% CI	*p*-Value
Viral load category	High-level vs. Moderate	1.305	0.803–2.123	0.283	1.205	0.757–1.917	0.432
Age	Continuous	1.009	0.986–1.032	0.466	0.999	0.970–1.030	0.934
Sex	Male vs. Female	1.226	0.769–1.954	0.393	1.170	0.730–1.880	0.499
Education	Higher vs. Lower	0.929	0.567–1.523	0.772	0.807	0.520–1.250	0.374
Socioeconomic barriers	Continuous	0.840	0.662–1.066	0.152	0.808	0.636–1.027	0.081

OR = crude odds ratio; aOR = adjusted odds ratio; CI = confidence interval. All variables were mutually adjusted in the multivariable model. Outcome: high absenteeism (11–15 days) versus lower absenteeism (5–10 days). Exposure: high-level versus moderate virological failure. The socioeconomic barrier score was entered as a continuous covariate in the original logistic regression model. It was entered categorically in the additional count-based analyses reported in Table 4.

**Table 4:** Adjusted count-based analyses of viral load and workplace absenteeism.

Analysis	Exposure	Adjusted IRR	95% CI	*p*-Value
Robust Poisson	High-level vs. moderate virological failure	1.05	0.99–1.11	0.123
Robust Poisson sensitivity analysis	log_10_ viral load *	1.10	1.02–1.19	0.011

IRR = incidence rate ratio; CI = confidence interval. * Viral load was log_10_-transformed for this sensitivity analysis; therefore IRR represents the relative change in the expected number of sick-leave days associated with a 10-fold increase in viral load. Both models were adjusted for age, sex, education level, and socioeconomic barrier score, with the barrier score entered categorically.

### 3.5. Count-Based and Continuous Viral-Load Sensitivity Analyses

Because absenteeism was originally measured as a count, additional analyses were conducted using the number of recorded sick-leave days without dichotomisation. In the adjusted robust Poisson model, high-level versus moderate virological failure was not significantly associated with the rate of sick-leave days (adjusted IRR = 1.05; 95% CI: 0.99–1.11; *p* = 0.123). In a further sensitivity analysis, viral load was modelled continuously on the log10 scale rather than using the study-defined 10,000-copy cut-point. Each 10-fold increase in viral load was associated with an approximately 10% higher rate of recorded sick-leave days (adjusted IRR = 1.10; 95% CI: 1.02–1.19; *p* = 0.011). Results of both count-based analyses are presented in Table 4.

## 4. Discussion

This study assessed whether high-level virological failure, compared with moderate virological failure, was associated with high workplace absenteeism among employed adults with HIV/PTB co-infection. Two descriptive findings were notable: 63.3% of this cohort selected for virological failure had viral load ≥10,001 copies/mL, and mean recorded sick leave was 10.05 days over three months. These findings demonstrate coexistence within the sample, not a causal or correlational relationship between virological status and absenteeism. The distribution of viral-load severity is clinically relevant, but because virological failure was an inclusion criterion, the 63.3% figure must not be interpreted as the prevalence of unsuppressed viral load in the source population. Persistent viral replication among people receiving ART may be associated with adherence difficulties, treatment interruption, drug resistance, treatment fatigue and access barriers [17,18,19], while HIV/TB co-treatment can add therapeutic complexity [3,4,20]. These mechanisms are contextual explanations from prior literature and were not directly tested in the present dataset.

The recorded absenteeism burden was substantial within this employed cohort: participants averaged about 10 sick-leave days in three months. The study measured absenteeism, not productivity, presenteeism, output or economic loss; therefore, no direct productivity effect can be inferred. Prior literature provides plausible pathways through illness episodes, treatment effects and healthcare attendance [5,8,9], but these mechanisms were not measured here. The multivariable analysis did not find evidence that high-level versus moderate virological failure was independently associated with high absenteeism (aOR = 1.205; 95% CI: 0.757–1.917; *p* = 0.432). The overall model was non-significant and explained only 3.0% of the variation in the dichotomised absenteeism outcome. This implies that an association was not detected within this sample under the conditions of this analysis. Several design and measurement features may have contributed to the null result. Viral load may relate only indirectly to work attendance, while TB severity, treatment adverse effects, healthcare utilisation, ART adherence, CD4 count, regimen and resistance, mental health, fatigue, substance use, stigma and workplace support may influence one or both variables [17,18,21]. These factors were not available for inclusion in the model, creating substantial potential for residual confounding and omitted-variable bias. The low Nagelkerke R^2^ is consistent with the limited explanatory coverage of the measured covariates and should temper interpretation of the adjusted estimates.

Selection is a central limitation. By design, the study included only people who remained formally employed while living with HIV/PTB and virological failure. Individuals who had left employment because of severe illness, disability, prolonged absence, hospitalisation, job loss or early retirement were not represented. This healthy-worker selection can reduce exposure and outcome variability and plausibly attenuate associations toward the null. The findings therefore do not generalise to unemployed people with HIV/PTB or to workers who had already exited employment because of illness. In addition, viral-load measurements and the three-month absenteeism window could not be precisely temporally aligned for every participant; the analysis cannot establish whether elevated viral load preceded absence, whether work constraints affected treatment access/adherence, or whether both reflected unmeasured causes. From a public health perspective, these findings highlight the need for integrated approaches that address both clinical and occupational dimensions of HIV/TB co-infection. While viral suppression remains a critical goal, improving workplace participation may require additional interventions beyond clinical management. These may include strengthened adherence support, improved access to care, and workplace-responsive strategies such as flexible scheduling, wellness programmes, and supportive sick-leave policies [8,9,18].

The contribution of this study is consequently narrow but relevant: it links routine virological data with recorded sick-leave information among employed adults with HIV/PTB co-infection and shows that viral-load severity alone did not explain high absenteeism in this dataset. The findings complement, rather than replace, the broader literature on work-related determinants of adherence and treatment outcomes.

### 4.1. Limitations

Several limitations should be considered. First, the restriction to currently employed adults with virological failure creates substantial selection bias and a healthy-worker effect: people who had already left work because of severe illness, disability, prolonged absence, hospitalisation, job loss or retirement were excluded, potentially attenuating associations and limiting generalisability to unemployed or work-exited populations. Second, the retrospective cross-sectional temporal structure precludes causal inference, and viral-load measurements could not always be aligned precisely with the preceding three-month absenteeism window. Third, the study-specific viral-load split at 10,000 copies/mL is not a WHO severity category, and categorisation may have reduced information compared with continuous/log-transformed viral load. Fourth, absenteeism was measured as absolute sick-leave days without consistent data on scheduled workdays, shift patterns or occupational categories; its binary cut-point (11–15 vs. 5–10 days) was study-specific and dichotomisation may have reduced power. Fifth, the structural/socioeconomic barrier score covered only a limited set of recorded domains and may have lacked sensitivity; zero recorded housing instability or food insecurity should not be interpreted as proof of absence. Sixth, key confounders, including ART adherence, CD4 count, ART regimen, drug resistance, TB severity, treatment duration, mental health, substance use, stigma, fatigue, and workplace support, were unavailable, leaving substantial residual confounding and omitted-variable bias. Seventh, the original prevalence-based sample-size calculation was not optimally aligned with the final regression question; the post hoc detectable-effect assessment indicates adequate power only for comparatively large effects (approximately OR 2.0), so modest associations may have been missed. Finally, the study was conducted in one sub-district and transferability to other occupational and epidemiological settings is limited.

This study compared absenteeism across levels of virological failure within an employed HIV/PTB co-infected cohort. High-level virological failure and substantial sick leave were both observed, but the adjusted analysis did not detect an association between viral-load category and high absenteeism. Given the healthy-worker effect, temporal ambiguity, limited exposure contrast, and unmeasured clinical and workplace determinants, the null finding should be interpreted cautiously and not as evidence that absenteeism is unrelated to health status.

This analysis adds evidence on the linkage between routine clinical markers and occupational absence, while also demonstrating the limits of viral load as a stand-alone explanatory variable for absenteeism. Future longitudinal studies should align viral-load and attendance time windows, include virally suppressed comparison groups, retain absenteeism as a count/rate with scheduled-work exposure, and measure treatment, TB-severity, and psychosocial and workplace determinants.

### 4.2. Public Health Implications

The concurrent occurrence of high levels of virological failure and substantial absenteeism in this selected cohort supports continued attention to integrated HIV/TB and occupational health services. However, because no independent association between viral-load category and high absenteeism was detected, the present data do not establish that improving viral suppression would itself reduce absenteeism or improve productivity. Workplace-responsive access to care and adherence support remain reasonable service considerations based on the broader evidence base [22] rather than a causal effect demonstrated by this study.

Given that the study population consisted of employed individuals, workplace-based health interventions may also play an important role. Employer-supported adherence strategies, such as flexible clinic attendance schedules and workplace health education programmes, may contribute to improved treatment adherence and reduced disease progression.

In addition, strengthening collaboration between public health programmes and occupational health services may improve health outcomes among employed individuals living with HIV and tuberculosis. Workplace-based screening, treatment support, and employee wellness programmes may provide opportunities for early identification of treatment challenges and improved adherence support.

### 4.3. Recommendations

Based on the findings of this study, several recommendations can be proposed.

First, healthcare programmes should continue evidence-based integrated HIV/TB management and adherence support.

Second, occupational-health services may facilitate clinic access and continuity of care through appropriate workplace policies.

Third, future research should use longitudinal designs, include suppressed and unsuppressed comparison groups, align exposure and outcome time windows, collect scheduled-work and shift data, and measure ART adherence, CD4 count, regimen, resistance, TB severity, mental health, stigma, fatigue, substance use, and workplace support.

## 5. Conclusions

Among 286 employed adults with HIV/PTB co-infection who were selected because they met criteria for virological failure, 63.3% had high-level virological failure (≥10,001 copies/mL) and mean sick leave was 10.05 days over three months. High absenteeism (11–15 days) occurred in 45.1%. The adjusted analysis did not detect an association between high-level versus moderate virological failure and high absenteeism. Accordingly, the study demonstrates the coexistence of these outcomes within the selected cohort, not correlation or causation. Interpretation is constrained by healthy-worker selection, lack of a virally suppressed comparison group, temporal ambiguity, study-specific categorisation of viral load and absenteeism, limited absenteeism context, and substantial residual confounding. The low explanatory value of the model suggests that important determinants of absenteeism were not captured. Longitudinal studies with aligned clinical and occupational measurements and broader clinical, behavioural, and workplace covariates are required to determine whether and how virological status relates to work absence. Further research using longitudinal designs and incorporating clinical and behavioural variables such as treatment adherence, regimen type, and drug resistance is recommended to better understand the determinants of viral load progression in HIV/TB co-infected populations.

## Figures and Tables

**Table 1:** Demographic characteristics, educational attainment, workplace absenteeism, and viral-load categories of participants.

Variable	Category	*n*	%
Sex	Male	141	49.3
	Female	145	50.7
Age group	20–29	67	23.4
	30–39	89	31.1
	40–49	79	27.6
	50+	51	17.8
Education	No schooling	3	1.0
	Primary	92	32.2
	Secondary	104	36.4
	Tertiary	87	30.4
Workplace absenteeism *	5–7 days	49	17.1
	8–10 days	108	37.8
	11–15 days	129	45.1
	>15 days	0	0.0
Viral Load Category	Moderate	105	36.7
	High	181	63.3

* Workplace absenteeism was measured as the number of sick-leave days taken during the preceding three months.

**Table 2:** Structural and socioeconomic barrier indicators.

Barrier Indicator	Coding/Summary	*n*	%
Employment-related barrier	Present	138	48.3
Other socioeconomic barrier	Present	132	46.2
Transport challenge	Recorded present	286	100.0
Housing instability	Recorded present	0	0.0
Food insecurity	Recorded present	0	0.0
Composite barrier score	Mean (SD), range 0–2	0.94 (0.99)	—

## Data Availability

The data underlying the findings of this study are available from the corresponding author upon reasonable request, subject to ethical and institutional restrictions in accordance with the Protection of Personal Information Act (POPIA), South Africa.

## Abbreviations

The following abbreviations are used in this manuscript:

aOR	adjusted odds ratio
ART	antiretroviral therapy
CD4	Cluster of Differentiation 4
CDC	Centers for Disease Control and Prevention
CI	confidence interval
Df	degrees of freedom
HIV	human immunodeficiency virus
OR	odds ratio
PLHIV	people living with HIV
PTB	pulmonary tuberculosis
SD	standard deviation
SPSS	Statistical Package for the Social Sciences
TB	tuberculosis
UJ	University of Johannesburg
UNAIDS	Joint United Nations Programme on HIV/AIDS
VL	viral load
WHO	World Health Organisation

## Nomenclature

Virological failure: Viral load ≥1000 copies/mL after the specified ART period. In the final analytic dataset, the observed minimum was 1200 copies/mL. The study-defined categories were moderate virological failure (1200–10,000 copies/mL) and high-level virological failure (≥10,001 copies/mL); Workplace absenteeism: Recorded sick-leave days during the preceding three months. For binary logistic regression, lower absenteeism was defined as 5–10 days and high absenteeism was defined as 11–15 days; HIV/TB co-infection: Refers to the simultaneous diagnosis of human immunodeficiency virus and pulmonary tuberculosis in the same individual; Employed individual: Refers to participants engaged in formal employment at the time of data collection; Opportunistic infections: Illnesses that occur more frequently and are more severe in people living with HIV; Workplace: A location where people perform tasks, jobs, and projects for their employer; Public health: The science of protecting and improving the health of people and their communities.
